# Use of House Cricket (*Acheta domesticus*) Powder in Yoghurt Products

**DOI:** 10.3390/foods13152426

**Published:** 2024-07-31

**Authors:** Kinga Karwacka, Adriana Łobacz, Justyna Ziajka, Anna Lis, Monika Małkowska-Kowalczyk, Maria Baranowska

**Affiliations:** Department of Dairy Science and Quality Management, Faculty of Food Science, University of Warmia and Mazury in Olsztyn, 10-709 Olsztyn, Poland; kinga.karwacka.1998@o2.pl (K.K.); justyna.ziajka@uwm.edu.pl (J.Z.); anna.lis@uwm.edu.pl (A.L.); monika.malkowska@uwm.edu.pl (M.M.-K.); mbb@uwm.edu.pl (M.B.)

**Keywords:** alternative protein sources, edible insects, *Acheta domesticus*, yoghurt with insect powder

## Abstract

This study aims to implement and increase values regarding the use of cricket powder in food. This is due to the need to increase the range of products enriched with cricket powder in order to increase the market and acceptance of insects in the diet. The work analyzed properties of yoghurt with cricket powder added (chemical composition, acidity, colour, consistency, degree of syneresis, texture and sensory analysis). Research has shown that the greater the addition of cricket powder, the higher the protein, fat and dry matter content and the lower the hardness of the yoghurt. As the content of cricket powder in yoghurt increased, the brightness (L*) and redness (a*) decreased, while the yellowness (b*) of the samples increased. A change in the colour of the yoghurt was observed with the increase in the cricket powder content; the yoghurt had a darker, browner colour. The best overall sensory rating compared to the control variant (6.4) was given to the yoghurt with 1.5% cricket powder added (5.7), while the worst rating was given to the yoghurt with 5% powder added (2 points out of 7).

## 1. Introduction

According to FAO projections, the world’s human population could reach 9.2 billion by 2050; accordingly, global food production will need to increase by 60%. To this end, it is important to explore new sources of nutrients, including, amongst others, protein(s). At the same time, it is important to conduct research into the diversity of food sources, and edible insects specifically can be a promising direction.

Potential barriers to insect consumption can be cultural, stemming from the belief that we are what we eat. This belief can hinder the acceptance of insects as part of the diet, as our perception of them as food is strongly tied to negative associations, which can outweigh their nutritional value. In order to convince consumers to eat insects on a regular basis, it is necessary to develop insect-infused products with an acceptable taste and form [1,2]. Greater acceptance can be achieved when insects are processed into known food powders, making the presence of this ingredient less noticeable. With an increasing number of papers published on the inclusion of insects in various food products, two main lines of research emerge.

One concerns the enrichment of products to increase their nutritional value, such as cakes, breads, pasta and cookies [3]. Insects are one of the projected trends in food consumption and production. They are currently treated as an alternative food source, but are predicted to become a major component of the diet of people around the world within the next few decades. The benefits of consuming insects are mainly due to their impact on economic, environmental, and nutritional aspects [4]. The EU register of new food products currently includes four different insect species, including the house cricket (*Acheta domesticus*)—the whole insect is available in frozen, dried, powdered and partially defatted powdered form [5]. In addition, the Commission Implementing Regulation (EU) 2023/5 of 3 January 2023, which relates to the approval of the house cricket (*Acheta domesticus*) as a novel food in the European Union, regulates the conditions under which crickets may be placed on the market as food, including labelling and safety requirements. This approval covers various forms of cricket processing, such as into flour and powder, and aims to ensure high standards of quality and consumer protection in the EU [6].

The house cricket has an average protein content of about 65% of its dry weight, which is higher than other common protein sources such as beef (50%), eggs (52%), milk (30%), and soy (45%). However, when comparing protein sources, it is essential to consider not only protein content but also bioavailability, as well as the mineral and vitamin content. Many traditional animal protein sources like beef, eggs, and milk provide a more complete nutrient profile, including essential vitamins and minerals which are vital for overall nutrition and health [7].

The other factor attracting attention in the context of insect protein hydrolysates is the production of bioactive peptides or biopeptides, which are involved in peptide–enzyme interactions, leading to a change in the enzyme’s formation and the inhibition of its metabolic activity. An example of bioactivity in house crickets is in oil and protein hydrolysate [8]. Fats are the second most important macronutrient fraction in edible insects, with unsaturated fatty acids (EFAs) present in higher percentages than saturated fatty acids (SFAs). House crickets contain 15–40% fat and essential fatty acids [8,9]. Chitin is a natural polysaccharide with antimicrobial and antioxidant properties; it is mainly derived from crustaceans, insects, and fungi. Chitin forms the epidermis and supports the exoskeleton of insects, and is a source of up to 50% of their carbohydrates [8,10].

The most studied functional and technological properties of edible insect proteins are stability, emulsification stability, soluble gel formation, water, and oil retention capacity [3]. On the Polish market, the leading group of food products are items containing entire insects, which account for 33% of the products offered. Whole insects are available in dried form with various flavour, for example, dried crickets with lime and chilli flavour. Another important product category is protein bars (23%), as well as powdered products (19%). Cricket powders are available in the form of powdered, ready-to-use mixes (e.g., for brownies or pancakes) and protein shakes in a variety of flavours [11]. Cricket powder has been added to the formulation of cereal products such as pasta and muffins. The use of insects resulted in a significant increase in the protein and mineral content of the resulting products [12]. An experiment was conducted to study the effect of adding grasshopper (*Oxya chinensis sinuosa*) to produce a high-protein yoghurt, exemplifying the incorporation of edible insects into diverse food products [13]. The present study aims to implement and increase the values regarding the use of cricket powder in food. This is due to the need to increase the range of products enriched with cricket powder to increase the market and acceptance of insects in the diet.

According to the IDF standard, yoghurt is a beverage made from standardized milk, which is thickened by the addition of skimmed milk powder or by evaporating some of the water, and then subjected to a pasteurization process and acidified with pure bacterial cultures [13]. The abundance of nutrients in yoghurt is due to the composition of the milk from which it is made. Dairy products are an excellent source of complete protein, milk fat, calcium, potassium, phosphorus, and many other essential nutrients. The high nutritional value of yoghurt depends on its chemical composition and the form of the ingredients, which facilitate their digestion, absorption, and assimilation. Milk and fermented beverages are dominated by casein and whey proteins in a ratio of 80/20. Natural yoghurts contain all the essential amino acids, with their amounts being higher than in the reference protein. The main energy component of milk and dairy products is fat, which has a wide variation in quality and quantity. More than 400 fatty acids have been identified in milk fat, and they occur in small amounts. Natural yoghurt is an excellent source of key macronutrients, such as calcium and phosphorus, which are essential in the daily human diet [14]. Calcium provided by dairy products has high bioavailability, which is mainly due to the presence of high protein, lactose, and vitamin D content in these products. Compared to milk, yoghurts have a lower pH value, which causes calcium to occur in an ionized form, which in turn increases its absorption through the intestines. Yoghurts are a rich source of calcium not only in terms of quantity (170 mg/100 g) but also in terms of bioavailability and calcium-to-protein ratio (39.5 mg of calcium per 1 g of protein) [15]. Fermented milk beverages are characterized by a mildly acidic taste. To increase the palatability of these products, manufacturers often introduce additives, such as sweeteners, fruits, nuts, flavourings, and an increase in milk solids [16].

The primary objective of this study was to investigate the feasibility of incorporating cricket powder into yoghurt formulations to enhance their protein content. Additionally, the study aimed to analyze the physicochemical properties and nutritional composition, conduct microbiological assessments, and perform sensory evaluations of the resultant products.

## 2. Materials and Methods

### 2.1. Yoghurt Production

The yoghurt was made from UHT milk (fat 2.11%, protein 3.32%, TS 10.99%), starter culture YC-C11(Chr. Hansen, Horsholm, Denmark), cricket powder—Cooking & Baking Cricket Protein Powder (SENS Foods, London, UK) with 70 g protein, 20 g fat, and 0.5 g carbohydrates per 100 g of product, and granulated skimmed milk powder (SM Gostyń, Poland) with protein content 35 g, fat 0.8 g, and carbohydrates 51 g per 100 g of product. The contents of milk powder and cricket powder in each variant are shown in Table 1.

The milk was heated to 42 °C and divided into 4 equal parts of 4 L each, and cricket powder and skimmed milk powder were added to each part as set out in Table 1 to achieve the dry matter content of the yoghurt at not less than 15%. The resulting mixture was reheated to 42 °C. Starter cultures of *Lactobacillus delbrueckii* subspecies *bulgaricus* and *Streptococcus thermophilus* were first dissolved in 200 mL of milk, and then 4 mL was added to each variant. The addition of 4 mL is based on the manufacturer’s data, which recommends the addition of 50 U of starter culture per 200 L of milk; accordingly, 4 mL of dissolved starter culture was used for 4 L of milk. After thorough mixing and pouring into sterile individual containers, the product was incubated at 42 °C for about 5–6 h. The incubation was terminated before the pH of the yoghurts exceeded 4.6. The course of acidification was measured using a multi-channel pH/pC/mV multiplexer (Cerko, Gdynia, Poland) equipped with ERH-13-6 type combination electrodes (HYDROMET, Gliwice, Poland). Measurements (in duplicate) were recorded at a frequency of every 5 min. After production, the yoghurts were stored at 4 °C for analyses performed on days 1, 7, 14, and 21.

### 2.2. Physicochemical and Rheological Analysis of Yoghurts

#### 2.2.1. Chemical Composition

The chemical composition of the UHT milk was determined using a MilkoScanTM FT2 (Foss, Hilleroed, Denmark), whereas the chemical composition of the yoghurts was carried out using a FoodScanTMLab analyzer (FOSS, Warsaw, Poland). A representative yoghurt sample was placed in a Petri dish and introduced into the measuring cuvette of the instrument. The analysis method was based on NIR transmittance in the wavelength range of 850–1100 nm (FoodScanTMLab user manual). A specially developed computer program calculated the average content and presented the percentage of each component in the yoghurt sample, covering protein, fat, and dry matter.

The pH was measured using the Elmetron pHmeter model CP-411, which was calibrated before the measurement.

A 0.25 M NaOH solution was utilized with a phenolphthalein indicator to titrate the milk and yoghurt samples (25 g yoghurt + 25 g water). The titration was carried out until a faint pink coloration, persisting for approximately 30 s, was observed. This measurement was performed in duplicate, following the guidelines of the Polish Standard.

#### 2.2.2. Analysis of Whey Syneresis

The whey syneresis was tested using two methods. The sieve method consisted of placing a sample of yoghurt (±20 g) on a sieve at ambient temperature and testing the leakage of the amount of whey after 2 h. The centrifuge method was applied by using a Thermo Scientific Heraeus Megafuge 16 centrifuge (Thermo Scientific, Waltham, MA, USA), where a tube filled with yoghurt (±20 g) was placed on it and then subjected to centrifugal force at 3300× *g* for 15 min at 10 °C. The results of both methods were expressed as percentages of leaked whey.

#### 2.2.3. Texture of Yoghurts

The texture of the yoghurts was determined using a texture analyzer TA.XTPlus Texture Analyser (Stable Micro Systems, Godalming, UK) using a 25 mm penetration test with an SMS P/25 aluminium cylindrical probe to a depth of 30 mm (i.e., 50% of the original height) and with a contact force of 0.098 N and a speed of 1.0 mm/s. Based on the obtained curves, the following characteristics were determined: firmness—expressed as the maximum penetration force [N]; consistency [N∙s]—expressed as the area of the curve measured to the point of maximum force (firmness) (range of positive loads recorded); cohesiveness [N]—expressed as the maximum force during probe return; and index of viscosity [N∙s]—expressed as the area of the negative area of the curve. The test was performed at 4 ± 1 °C. The texture determination of the yoghurt was carried out in duplicate.

#### 2.2.4. Assessment of Colour

Instrumental colour analysis was carried out using a CM-3500d spectrophotometer (Konica Minolta, Tokyo, Japan). Reflectance spectrophotometry in the visible light range (from 400 to 700 nm) was used to record the spectrum of the yoghurt samples. Reflectance was measured by placing a Petri dish with the test sample on an aperture mask (CM-A122) with a field diameter of 8 mm in such a way that the aperture’s measurement field remained completely covered. The surface of the sample was illuminated with diffuse light at an angle of 8° (d/8) with respect to the normal to the surface of the test material and was then reflected and scattered. The light source used in the colour measurements was a D65 illuminant and a colorimetric observer with a field of view of 10°. The analysis was carried out using CM-S100w SpectraMagicTM NX Lite Version 2.3 software. The instrument was calibrated with a white (CM A120) and black (CM A124) standard before the test. The colour parameters were determined based on the CIE Lab model, in which colour was represented by an achromatic component L*—colour brightness index on a scale from 0 (black) to 100% (white)—and two chromatic components: a*—red colour index on a scale from −60 (green colour) to 60 (red colour) and b*—yellow colour index on a scale from −60 (blue colour) to 60 (yellow colour). The colour of each yoghurt sample was measured in five replicates.

#### 2.2.5. Assessment of Viscosity

The yoghurt samples were monitored by performing a time–curing test in oscillation by means of a Physica MCR 102 rheometer (Anton Paar GmbH, Graz, Austria), controlled by the RheoCompass software (v. 1.31.69, Anton Paar, Graz, Austria). Each sample (20 mL) was poured into the concentric cylinders (CC27) of the rheometer and the test was performed for 200 min at 42.0 ± 0.1 °C, applying constant strain (1%) and frequency (1 Hz) values. The storage modulus (G′), loss modulus (G″), and loss tangent (tan δ = G″/G′) were measured and recorded each 10 s.

#### 2.2.6. Microstructure of Yoghurts

The structure of the yoghurts was determined using an FEI Type Quanta 200 scanning microscope (FEI Company, Hillsboro, OR, USA). The yoghurts were observed in their natural state in ESEM mode after carefully applying the sample to a special module designed for use on a Petri table in the microscope and freezing in a chamber to −10 °C. A Dual BSD detector at 100× magnification, 1.02 mm image size, 30,000 kV electrode accelerating voltage, and 500 um scale was used.

### 2.3. Microbiological Analysis

To determine the total viable count (TVC), the classic plate method was used with the surface culture on CASO (Merck, Germany) microbiological medium prepared according to the manufacturer’s instructions (chemical composition: casein peptone, soy flour peptone, sodium chloride, agar-agar). Decimal dilutions were made of each yoghurt variant using 9 mL of sterile peptone water. One dilution was inoculated onto three plates by taking 0.1 mL for each and spreading it evenly on the medium using sterile strokes. The plates were then incubated under aerobic conditions at 37 °C for 48 h. Plates with grown microbial counts between 30 and 300 were selected for counting. The number of bacteria colony-forming units (L) was calculated using the following formula:

$$L=C\times d\times a[\frac{cfu}{mL}]$$

where:

C—sum of colonies on all plates selected for counting;

d—dilution index;

a—the ratio of the amount of material held.

### 2.4. Sensory Analysis

The analytical part of the experiment included a sensory analysis of the tested products, carried out by a team of 10 evaluators. Four distinguishing characteristics, including appearance (colour, syneresis, attraction, whey leakage), aroma (yoghurt, sour, atypical), taste (sweet, sour, yoghurt, bitter, atypical), and texture (ductile, uniform, dense, fine liquid, sandy, gross, smooth) were evaluated by a sensory panel where 1 was no feature and 7 was the strong presence of a feature.

### 2.5. Statistical Analysis

The results obtained were subjected to statistical analysis. Mean values and standard deviations were calculated, and statistically significant differences between means were estimated using one-way analysis of variance (ANOVA) and Tukey’s test (*p* ≤ 0.05). Calculations were performed using the statistical package StatSoft Inc. Statistica v. 13.1 software (Tulsa, OK, USA).

## 3. Results and Discussion

### 3.1. Nutrient Composition

It was noticed that the greater the addition of cricket powder, the higher the protein, fat, and dry matter content of the yoghurt (Table 2). Yoghurt with 5% cricket powder addition had the highest protein (7.83 ± 0.05%) and fat contents (5.18 ± 0.07%). The addition of cricket powder increased the protein and fat contents of the products. Cricket powder is a source of protein (70 g/100 g powder), and it contains significant amounts of fat (20 g/100 g powder). Edible insects are an excellent source of nutrients, such as minerals that provide B complex, proteins, vitamins, and lipids with a lower ratio of omega-6 to omega-3. A paper was published [9] in which the addition of cricket powder to chocolate cookies was investigated and, based on the results obtained, it was concluded that the addition of cricket powder modified the nutritional composition of chocolate cookies. The addition of cricket powder increased the moisture and protein content and reduced the ash, fat and carbohydrate content. Currently, global trends in product development favour high protein and ash content and low fat and carbohydrate content [8,9,17].

### 3.2. Acidity Profile during Fermentation

**Figure 1:**
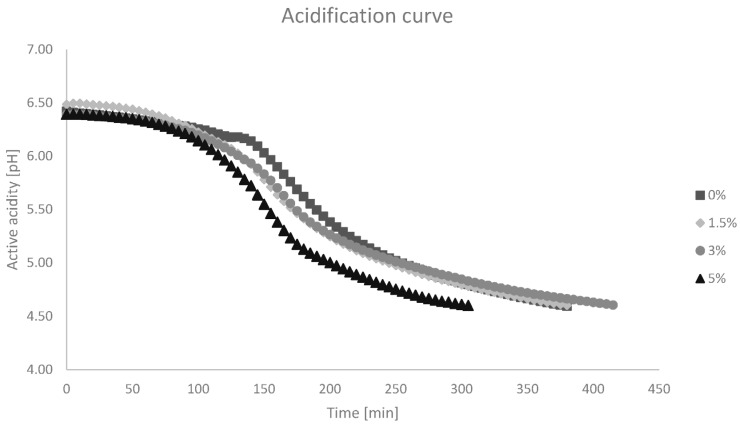
Yoghurt acidification curve. Explanatory notes: 0%—control yoghurt [0% added cricket powder]; 1.5%—yoghurt with 1.5% added cricket powder; 3%—yoghurt with 3% added cricket powder; and 5%—yoghurt with 5% added cricket powder.

Figure 1 shows the acidification curve of the different variants of produced yoghurt. Yoghurt with 5% cricket powder addition reached pH 4.6 the fastest (305 min), while yoghurt with 3% cricket powder addition was incubated the longest (415 min). The variant with the addition of 1.5% cricket powder completed incubation at 380 min, the same as the control yoghurt.

### 3.3. Profile of Yoghurt Acidity during Storage

The pH value in yoghurt should be in the range of 4–4.5 [18]. The obtained initial pH value for variants of produced yoghurt was 4.6. The decrease in pH during storage was 0.4 for the variant with the addition of 1.5% cricket powder, and reached a final value on the 21st day of storage, 4.19, and for the variant with 3% addition, the final value was 4.25. In the control variant (0% cricket powder addition) and with 5% cricket powder addition, the decrease in pH was 0.3, reaching a final value of 4.3. In all variants, there was a decrease in pH values between days 1 and 21, indicating normal activity of the bacteria that were used as starter culture. In a study by Bartkiene et al. (2023), the addition of cricket powder to bread was tested It was found that the addition of cricket powder in breadmaking resulted in a decrease in pH. After 24 h of fermentation, the pH of cricket powder dropped by an average of 13.0%, and after 48 h of fermentation by an average of 28.0% [19].

For all variants of yoghurts, an increase in titratable acidity was observed, analogous to pH; the highest value (58.8 ± 0.1 °SH) compared to the control variant (57.6 ± 0.2 °SH) was obtained for the yoghurt with 1.5% addition of cricket powder, while the lowest value (56.6 ± 0.1 °SH) was obtained in the case of yoghurt with a 5% addition of the cricket powder. Kalicka et al. (2015) studied the acidity of peach yoghurts and found that acidity was in the range of 28.12–35.60 °SH. For natural yoghurts, the values should be in the range of 44.4–61.2 °SH, depending on the protein and fat content [20].

### 3.4. Assessment of Viscosity

Table 3 shows the results of the effect of fermentation at 42 °C on the viscoelastic properties of the produced yoghurts (0, 1.5, 3, and 5% cricket powder). For the control sample (0% added cricket powder), the onset of curd formation (tan δ = G″/G′ = 1) was at 166.5 ± 3.5 min. The point of intersection of the curves describing storage modulus (G′) and loss modulus (G″) on the graph marks the beginning of curd formation. The addition of cricket powder in each variant tested reduced the time of clot formation: 143.5 ± 5.0 (1.5% cricket powder addition), 119.5 ± 0.7 (3% cricket powder addition), and 128.5 ± 0.7 (5% cricket powder addition).

The product retains the characteristics of a viscoelastic solid if the storage modulus (G′) is greater than the loss modulus (G″), G′ > G″; the inverse relationship (G′ < G″) is said to be a viscoelastic liquid [20]. The resulting curd in each variant showed viscoelastic properties (G′ > G″). The highest loss factor value, for a time of 200 min, was obtained for the sample with 1.5% cricket powder addition, 0.405 (Table 4), so it was the most compact yoghurt. The lowest value of loss factor, the least compact yoghurt, was obtained for the sample with 5% addition of cricket powder: 0.337.

### 3.5. Microstructure of Yoghurts

**Figure 2:**
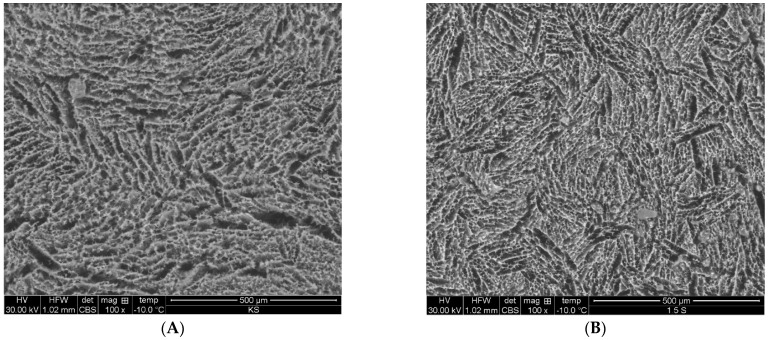

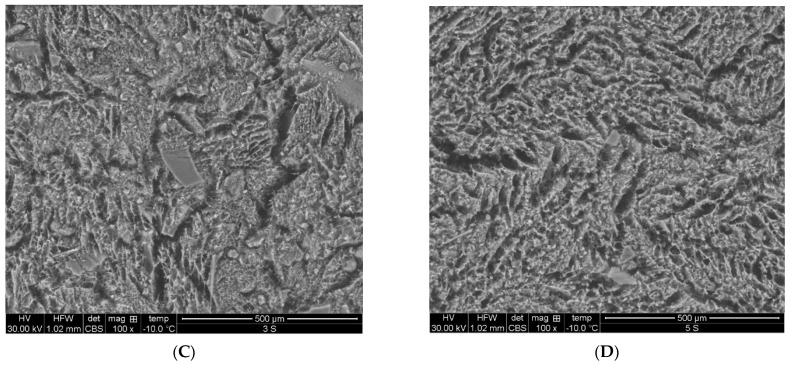
Microstructure of (**A**) control yoghurt with 0% cricket powder addition, (**B**) yoghurt with 1.5% cricket powder addition, (**C**) yoghurt with 3% cricket powder addition, and (**D**) yoghurt with 5% cricket powder addition.

Electron microscopy studies have shown differences in microstructure between particles of the control yoghurt (0% cricket powder) and yoghurts with cricket powder added. Images of yoghurts with added cricket powder show sharp irregular-shaped elements; the structure appears loose (Figure 2). Changes in the microstructure between samples with the addition of cricket powder and the control sample result from changes in the characteristics of the particles. The structure of the control yoghurt was a hard and bound gel. Additionally, the gel appeared to have regular-length casein fibres. On the other hand, yoghurts with cricket powder added showed inconsistency, a disconnected gel, and a matrix containing large pores and spaces. The yoghurt gel with powder added had an irregular shape, with short casein fibres and individual casein fibres [21].

### 3.6. Syneresis of Whey

Whey syneresis in yoghurts was measured using two methods: the centrifuge method (Table 5a) and the sieve method (Table 5b).

Considering the results shown in Table 5a,b, it is noticeable that there are significant differences between the sieve method and the centrifuge method. The centrifuge method is the more precise method due to the effect of centrifugal force. An increase in the yoghurt’s susceptibility to syneresis was observed during sample storage, which became particularly apparent with the centrifuge method [22,23]. The degree of syneresis assessed by the sieve method did not coincide with the data obtained by the centrifugal method. However, the analogy of the increase in whey syneresis with storage time and with the addition of house cricket powder in both methods was noticeable. The curd stabilization of fermented milk beverages with flavourings can be improved by increasing the milk solids or by adding non-dairy stabilizers and thickeners [24]. Good-quality yoghurt should be characterized by thick and smooth curds with no whey leakage. In the case of a thermostatically produced product, the occurrence of intense syneresis on the surface of the yoghurt is associated by consumers with poor quality, even if there is information about the naturalness of such a process [24]. The greatest differences in syneresis between samples were observed after 14 days of production, especially between the control yoghurt (0% cricket powder) and the yoghurt containing 5% cricket powder (*p* < 0.05). After 21 days of storage, yoghurt with 5% cricket powder addition had a whey flow of 56%. Increasing the proportion of milk proteins contributes to increasing the compactness of the curd by increasing particle packing and water absorption to prevent adverse changes that may occur during the transport or storage of products. Traditionally, the addition of skimmed milk powder was used for this purpose [25]. Whey syneresis was the lowest in the control yoghurt, due to the highest addition of skimmed milk powder.

Whey syneresis can be reduced using various natural methods; for example, the addition of linoleic acid (LA) to yoghurt reduces whey syneresis. LA interacts with milk proteins such as casein (CS), β-lactoglobulin (β-Lg), and bovine serum albumin (BSA), changing their structure. This creates a more uniform and compact microstructure of the yoghurt, which reduces the release of whey. The addition of natural thickeners such as xanthan gum, carrageenan, or inulin, can improve the water-binding capacity of yoghurt, thereby reducing whey syneresis. Yoghurt enriched in CLA by fermentation with lactic acid bacteria (LAB) showed reduced whey syneresis. CLA has antioxidant and anticarcinogenic properties, which contributes to the improved quality of yoghurt. The addition of plant extracts such as rose (Rosa rugosa) or date (Phoenix dactylifera) extract improves the water-binding capacity and reduces whey syneresis. These extracts form stable complexes with casein, which helps to retain whey in the yoghurt matrix. These natural methods not only reduce whey syneresis, but can also improve the nutritional value and sensory properties of yoghurt [26].

### 3.7. Texture of Yoghurts

Texture, next to taste and smell, is one of the most important factors in yoghurt quality. Defects in the texture of yoghurt are the main reason for the lower consumer acceptance of this group of dairy products. The most common texture defects include a loose consistency of yoghurt produced using the thermostatic method [27]. The increase in the hardness of yoghurts with the addition of whey protein concentrates can be explained by the correlation of the content of casein and whey proteins. When the casein-to-whey-protein ratio decreases, the hardness of the yoghurt gel increases [27]. With the passage of storage time, the hardness of yoghurts in each variant increases, but these results are not statistically significant (*p* < 0.05). However, the greater the addition of cricket powder, the lower the hardness of the yoghurts (Table 6). The lowest hardness was observed in yoghurts with 5% cricket powder added (0.61 ± 0.02 N), compared to the control variant (1.18 ± 0.09 N). In chocolate chip cookie research, the addition of cricket powder in the production of chocolate chip cookies resulted in a significant reduction in the hardness of the cookies [28].

The consistency of the yoghurts remained constant during storage. The higher the addition of cricket powder, the looser the consistency of the yoghurts. Statistically significant differences (*p* < 0.05) were found between yoghurts with the addition of 3% and 5% cricket powder and the control yoghurt. In the case of the index of viscosity, a significant increase in the value of the parameter was observed in yoghurts with the addition of 1.5% cricket powder compared to the control variant without the addition of powder. The inclusion of cricket powder in the production of the chocolate cookie resulted in an increase in the final viscosity of the product [28]. Cricket powder, both defatted and non-skimmed, has a high protein content ranging from 70.51% to 76.02%. It also has good water and oil binding capacity, as well as a high foaming capacity and foam stability. Cricket powder can be a valuable food ingredient, improving its nutritional value and sensory properties [29].

The cohesion of the obtained gels, describing the strength of internal bonds holding the product together, decreased with the addition of cricket powder. With the passage of time of storage of the control yoghurts and those with the addition of 1.5% cricket powder, an increase in consistency was observed, but the results were not statistically different (*p* < 0.05). In the case of yoghurts with 3% and 5% cricket powder added, this parameter did not change significantly. Cohesion values range from 0 to 1, where 0 means that the sample does not return to its original shape after deformation, as is the case with gels, and 1 means complete reconstruction, e.g., in the case of liquids [30]. In the case of the tested yoghurts, negative values were obtained, indicating that the tested sample does not return to its original shape.

### 3.8. Assessment of Colour Parameters

The results of the analysis of colour parameters of the produced yoghurts in terms of L*, a*, and b* values can be found in Table 7. As the content of cricket powder in yoghurts increased, the brightness (L*) and redness (a*) decreased, while the yellowness (b*) of the samples increased. A change in the colour of the yoghurts was observed with the increase in the cricket powder content; the yoghurts had a darker, browner colour. In studies conducted on chocolate cookies with the addition of cricket powder, a change in colour was also observed [29]. Another study also found that cricket protein in crackers had a negative effect on colour assessment [31]. In the study on pancakes with the addition of cricket powder, all pancakes with the addition of insect powder were darker than the control sample (increase in the value of the L* parameter), and the greater the addition of each type of cricket powder, the darker the colour [18].

### 3.9. Microbiological Analysis

Table 8 shows the level of total place count present in obtained yoghurts during storage. In each variant, an increase in the number of microorganisms was observed with time. Comparable values were reached above 8.15 log cfu/mL (control yoghurt) to 8.46 log cfu/mL (yoghurt with 5% cricket powder addition). The Polish Standard specifies the requirements for the number of live microbial cells that are to be present in fermented milk drinks throughout their declared shelf life. According to these recommendations, the number of characteristic microflora should be at least 10^7^ cfu in 1 g, while the number of additional microflora should be at least 10^6^ cfu in 1 g of the product [32]. In light of the given requirements, the number of microflora is considered high.

### 3.10. Sensory Analysis

The sensory analysis of the produced yoghurts was carried out after 1, 7, 14, and 21 days of storage. The samples for sensory evaluation directly before the analyses were standardized, so such characteristics as delamination and whey leakage were not evaluated. According to the evaluators, the colour of the yoghurts did not change during storage. The colour of yoghurt with 5% cricket powder addition was rated the worst (4.3), while the colour of yoghurt with 1.5% cricket powder addition was rated the best (6.4) compared to the control variant (7). Yoghurt with 1.5% cricket powder addition proved to be the most attractive product for the evaluators in terms of appearance.

As storage time increased, the smell and taste of the yoghurt became less and less noticeable; the evaluation of this parameter also decreased as the addition of cricket powder increased. However, the sour taste and odour intensified during refrigerated storage of the yoghurt. Yoghurt with the 1.5% addition of cricket powder had the highest intensity of these two characteristics. As the addition of cricket powder increased (0, 1.5, 3, and 5%), the intensity of the foreign, bitter taste and odour increased (1, 2, 5. 3, 6, respectively).

One of the most important parameters for the sensory evaluation of yoghurts is consistency. Consumers pay special attention to it when purchasing a product [33]. According to the definition, consistency is defined as the degree of density, viscosity, and compactness of a product or its specific ingredients, characterizing certain texture features [34]. The degree of consistency formation of yoghurts is influenced by the introduction of stabilizing and thickening additives [35]. The quality of yoghurt consistency is influenced by the content of dry matter ingredients [36].

The texture of the analyzed yoghurts was not rated as lumpy or sandy. As the addition of cricket powder increased (0, 1.5, 3, and 5%), the texture of the yoghurts became more fluid and malleable (1.8, 3.5, 5.3, and 7, respectively), according to the evaluators.

The yoghurt with 1.5% cricket powder addition received the best (5.7) overall rating compared to the control variant (6.3), while the yoghurt with 5% powder addition received the worst rating (2). Bartkowicz [18] assessed three samples of a cocktail based on bilberries with the addition of various amounts of ground edible house cricket *Acheta domesticus* (1.0%, 2.9%, and 4.8%). The cocktail with 2.9% cricket powder received the highest consumer acceptance. More than half (53.5%) of respondents would willingly consume cocktails in the form presented in the consumer sensory evaluation. Moreover, 8.8% of consumers had a negative attitude towards the presented cocktail and 37.7% were undecided. The respondents also commented on the health benefits of the evaluated product. Nearly half of the respondents (49.1%) indicated “rather large” and 11.4% indicated “large” health benefits. Only 5.3% of the surveyed group indicated “none” and “small benefits”, and 10.5% “rather small”, and 23.7% were undecided.

## Figures and Tables

**Table 1:** The contents of cricket powder and milk powder in different variants of yoghurt.

Variant of Yoghurt	Cricket Powder (%)	Skimmed Milk Powder (%)
A	0	5
B	1.5	3.5
C	3	2
D	5	0

**Table 2:** The chemical composition of milk and manufactured yoghurts.

Variant	Protein [%]	Fat [%]	Dry Matter [%]
milk	3.33 ± 0.03	2.00 ± 0.04	11.14 ± 0.05
control yoghurt [0% added cricket powder]	5.32 ± 0.02	2.08 ± 0.01	15.67 ± 0.08
yoghurt with 1.5% added cricket powder	6.02 ± 0.06	2.76 ± 0.03	16.20 ± 0.06
yoghurt with 3% added cricket powder	6.48 ± 0.01	3.53 ± 0.06	18.43 ± 0.03
yoghurt with 5% added cricket powder	7.83 ± 0.05	5.18 ± 0.07	19.87 ± 0.05

**Table 3:** Average values (±standard deviation) of time, storage modulus, and loss modulus for each variant during clot formation.

	Curing|Sol–Gel Point|G′, G″ (t)			
Variant	Time [min]	Storage Modulus [Pa]	Loss Modulus [Pa]	Loss Factor [-]
Control yoghurt [0% added cricket powder]	166.5 ± 3.5	0.0566905 ± 0.01	0.0566905 ± 0.01	1
Yoghurt with 1.5% added cricket powder	143.5 ± 5.0	0.0979685 ± 0.00	0.0979685 ± 0.00	1
Yoghurt with 3% added cricket powder	119.5 ± 0.7	0.0799965 ± 0.00	0.0799965 ± 0.00	1
Yoghurt with 5% added cricket powder	128.5 ± 0.7	0.100281 ± 0.00	0.100281 ± 0.00	1

**Table 4:** Mean values (±standard deviation) of storage modulus, loss modulus, and loss factor at 200 min for each variant of produced yoghurt.

	Curve|Maximum			
Variant	Time [min]	Storage Modulus [Pa]	Loss Modulus [Pa]	Loss Factor [-]
Control yoghurt [0% added cricket powder]	200 ± 0.0	34.6045 ± 8.30	13.7665 ± 3.54	0.396 ± 0.01
Yoghurt with 1.5% added cricket powder	200 ± 0.0	96.367 ± 5.13	39.075 ± 1.59	0.405 ± 0.00
Yoghurt with 3% added cricket powder	200 ± 0.0	68.9305 ± 0.24	25.8495 ± 0.03	0.375 ± 0.00
Yoghurt with 5% added cricket powder	200 ± 0.0	81.989 ± 2.29	27.641 ± 0.98	0.337 ± 0.00

**Table 5:** (**a**) Whey syneresis of yoghurts—the centrifuge method. (**b**) Whey syneresis of yoghurts—the sieve method.

(a)				
	Level of Cricket Powder Addition to Yoghurt			
Day	0 (%)	1.5 (%)	3 (%)	5 (%)
1	23.41 ^dx^ ± 0.48	23.61 ^dx^ ± 0.49	33.54 ^cw^ ± 2.06	34.63 ^dw^ ± 1.21
7	30.18 ^cx^ ± 0.36	31.36 ^cyx^ ± 0.17	34.96 ^cy^ ± 1.07	39.79 ^cw^ ± 0.36
14	34.06 ^by^ ± 0.53	47.11 ^bx^ ± 0.23	48.33 ^bwx^ ± 0.26	49.39 ^bw^ ± 0.62
21	43.69 ^ay^ ± 1.20	51.45 ^ax^ ± 0.07	54.15 ^awx^ ± 0.23	56.04 ^aw^ ± 0.65
**(b)**				
	**Level of Cricket Powder Addition to Yoghurt**			
**Day**	**0 TM (%)**	**1.5 TM (%)**	**3 TM (%)**	**5 TM (%)**
1	20.25 ^ax^ ± 1.09	17.20 ^ax^ ± 0.87	27.14 ^aw^ ± 2.88	31.72 ^aw^ ± 0.09
7	20.66 ^aw^ ± 0.33	20.49 ^aw^ ± 0.07	29.63 ^aw^ ± 1.50	31.94 ^aw^ ± 5.89
14	21.75 ^ax^ ± 0.30	20.96 ^ax^ ± 2.73	29.98 ^aw^ ± 1.62	32.38 ^aw^ ± 1.38
21	22.59 ^ax^ ± 0.79	21.58 ^ax^ ± 1.11	33.22 ^aw^ ± 0.27	34.54 ^aw^ ± 0.39

Explanatory notes: 0%—control yoghurt [0% added cricket powder], 1.5%—yoghurt with 1.5% added cricket powder, 3%—yoghurt with 3% added cricket powder, 5%—yoghurt with 5% added cricket powder. ^w–y^ average values in the same row marked with different symbols are statistically significantly different (*p* < 0.05); ^a–d^ the mean values in the same column indicated by different symbols are statistically significantly different (*p* < 0.05). Explanatory notes: 0%—control yoghurt [0% added cricket powder], 1.5%—yoghurt with 1.5% added cricket powder, 3%—yoghurt with 3% added cricket powder, 5%—yoghurt with 5% added cricket powder. ^w,x^ average values in the same row marked with different symbols are statistically significantly different (*p* < 0.05); ^a^ the mean values in the same column indicated by different symbols are statistically significantly different (*p* < 0.05).

**Table 6:** Texture parameters of the produced yoghurts with added cricket powder.

	Cricket Powder Addition [%] to Yoghurt	Day			
		1	7	14	21
Firmness (N)	0%	1.18 ^aw^ ± 0.09	1.24 ^aw^ ± 0.01	1.32 ^aw^ ± 0.07	1.39 ^aw^ ± 0.05
	1.5%	1.24 ^aw^ ± 0.05	1.17 ^aw^ ± 0.08	1.20 ^aw^ ± 0.01	1.26 ^aw^ ± 0.02
	3%	0.76 ^ax^ ± 0.04	0.76 ^ax^ ± 0.03	0.81 ^ax^ ± 0.01	0.77 ^ax^ ± 0.01
	5%	0.61 ^ax^ ± 0.02	0.63 ^ax^ ± 0.00	0.65 ^ay^ ± 0.02	0.61^ay^ ± 0.05
Consistency (N·s)	0%	27.12 ^aw^ ± 1.05	28.87 ^aw^ ± 0.12	29.62 ^aw^ ± 1.71	30.58 ^aw^ ± 0.05
	1.5%	25.32 ^aw^ ± 1.22	25.83 ^aw^ ± 1.97	26.49 ^aw^ ± 0.28	28.80 ^aw^ ± 0.30
	3%	15.70 ^ax^ ± 1.23	16.60 ^ax^ ± 0.21	17.20 ^ax^ ± 0.60	16.20 ^ax^ ± 0.22
	5%	12.42 ^ax^ ± 0.69	12.70 ^ax^ ± 0.28	12.92 ^ay^ ± 0.56	11.96 ^ay^ ± 0.98
Cohesiveness (N)	0%	−0.70 ^bw^ ± 0.04	−0.79 ^abw^ ± 0.03	−0.80 ^abw^ ± 0.03	−0.84 ^aw^ ± 0.03
	1.5%	−0.67 ^aw^ ± 0.07	−0.72 ^ax^ ± 0.02	−0.72 ^ax^ ± 0.01	−0.82 ^ax^ ± 0.04
	3%	−0.41 ^ax^ ± 0.02	−0.38 ^ay^ ± 0.01	−0.37 ^ay^ ± 0.01	−0.39 ^ay^ ± 0.01
	5%	−0.25 ^ax^ ± 0.01	−0.25 ^az^ ± 0.00	−0.25 ^az^ ± 0.01	−0.24 ^az^ ± 0.02
Index of Viscosity(N·s)	0%	−0.43 ^bx^ ± 0.14	−0.33 ^bw^ ± 0.04	−0.36 ^bw^ ± 0.02	−1.89 ^aw^ ± 0.05
	1.5%	−1.54 ^aw^ ± 0.21	−1.65 ^bw^ ± 0.05	−1.70 ^bw^ ± 0.08	−0.39 ^bx^ ± 0.01
	3%	−0.90 ^ax^ ± 0.09	−0.59 ^aw^ ± 0.50	−0.49 ^aw^ ± 0.44	−0.18 ^ax^ ± 0.05
	5%	−0.52 ^ax^ ± 0.05	−0.34 ^aw^ ± 0.29	−0.35 ^aw^ ± 0.25	−0.33 ^ax^ ± 0.21

Explanatory notes: ^w–z^ average values in the same column marked with different symbols are statistically significantly different (*p* < 0.05); ^a,b^ the mean values in the same row indicated by different symbols are statistically significantly different (*p* < 0.05).

**Table 7:** Colorimetric properties of produced yoghurts with addition of cricket powder during storage.

	Cricket Powder Addition [%] to Yoghurt	Day			
		1	7	14	21
L*	0%	86.06 ^az^ ± 0.05	85.08 ^ay^ ± 0.08	85.61 ^ax^ ± 0.15	88.64 ^aw^ ± 0.28
	1.5%	80.56 ^bx^ ± 0.18	80.61 ^bx^ ± 0.29	79.82 ^by^ ± 0.09	83.85 ^bw^ ± 0.21
	3%	64.89 ^cz^ ± 1.08	63.02 ^cy^ ± 0.24	67.06 ^cx^ ± 0.48	70.88 ^cw^ ± 0.52
	5%	64.78 ^cx^ ± 0.15	61.01 ^dy^ ± 0.50	65.27 ^dx^ ± 0.32	69.68 ^dw^ ± 0.50
a*	0%	−3.25 ^by^ ± 0.02	−3.31 ^dx^ ± 0.03	−3.29 ^dxy^ ± 0.03	−3.39 ^dw^ ± 0.02
	1.5%	−1.27 ^cy^ ± 0.04	−1.52 ^cx^ ± 0.08	−1.37 ^cy^ ± 0.03	−1.69 ^cw^ ± 0.08
	3%	0.75 ^dx^ ± 0.25	1.28 ^bw^ ± 0.10	0.75 ^bx^ ± 0.06	0.39 ^by^ ± 0.05
	5%	1.16 ^ax^ ± 0.06	1.51 ^aw^ ± 0.10	1.00 ^ay^ ± 0.07	0.61 ^az^ ± 0.03
b*	0%	8.96 ^by^ ± 0.05	8.87 ^dy^ ± 0.08	9.22 ^cx^ ± 0.02	9.52 ^cw^ ± 0.08
	1.5%	8.95 ^bx^ ± 0.20	9.26 ^cw^ ± 0.10	9.08 ^cwx^ ± 0.14	9.28 ^cw^ ± 0.10
	3%	9.49 ^bx^ ± 0.84	10.13 ^bxy^ ± 0.12	11.82 ^aw^ ± 0.43	10.99 ^bwx^ ± 0.06
	5%	12.06 ^aw^ ± 0.19	11.24 ^ax^ ± 0.21	10.22 ^by^ ± 0.22	12.42 ^aw^ ± 0.26

Explanatory notes: ^w–z^ average values in the same row marked with different symbols are statistically significantly different (*p* < 0.05); ^a–d^ the mean values in the same column indicated by different symbols are statistically significantly different (*p* < 0.05).

**Table 8:** Total viable count (TVC) in yoghurts during storage.

Variant/Time [Days]	TVC (log cfu/mL)			
	1	7	14	21
control yoghurt (0% cricket powder addition)	7.25 ± 0.04	7.16 ± 0.66	7.77 ± 0.03	8.15 ± 0.05
Yoghurt with 1.5% added cricket powder	7.47 ± 0.14	8.02 ± 0.36	8.18 ± 0.11	8.42 ± 0.05
Yoghurt with 3% added cricket powder	7.12 ± 0.19	7.21 ± 0.19	8.05 ± 0.02	8.42 ± 0.04
Yoghurt with 5% added cricket powder	7.50 ± 0.02	7.55 ± 0.06	7.78 ± 0.13	8.46 ± 0.01

## Data Availability

The original contributions presented in the study are included in the article, further inquiries can be directed to the corresponding author.
